# Supplementary material for: AURKA controls oocyte spindle assembly checkpoint and chromosome alignment by HEC1 phosphorylation
Source: Life Sci Alliance. 2025 May 6;8(7):e202403146. doi: 10.26508/lsa.202403146 (PMC12056248; doi:10.26508/lsa.202403146)
Supplement: Supplementary file 1 [file LSA-2024-03146_TableS1.docx]

|  | Phosphorylation site | | |
| --- | --- | --- | --- |
| Comparison | pSer44 | pSer55 | pSer69 |
| WT vs. A-KO | n.s. | n.s. | **** |
| WT vs. B-KO | n.s. | n.s. | n.s. |
| WT vs. C-KO | n.s. | *** | n.s. |
| WT vs. AB-KO | n.s. | n.s. | **** |
| WT vs. AC-KO | **** | **** | **** |
| WT vs. BC-KO | n.s. | n.s. | ** |
| A-KO vs. B-KO | n.s. | n.s. | **** |
| A-KO vs. C-KO | n.s. | ** | **** |
| A-KO vs. AB-KO | n.s. | n.s. | n.s. |
| A-KO vs. AC-KO | **** | *** | ** |
| A-KO vs. BC-KO | n.s. | n.s. | **** |
| B-KO vs. C-KO | n.s. | * | n.s. |
| B-KO vs. AB-KO | n.s. | n.s. | **** |
| B-KO vs. AC-KO | **** | *** | **** |
| B-KO vs. BC-KO | n.s. | n.s. | n.s. |
| C-KO vs. AB-KO | n.s. | *** | **** |
| C-KO vs. AC-KO | **** | n.s. | **** |
| C-KO vs. BC-KO | n.s. | n.s. | n.s. |
| AB-KO vs. AC-KO | **** | **** | ** |
| AB-KO vs. BC-KO | n.s. | n.s. | **** |
| AC-KO vs. BC-KO | *** | ** | **** |

Table S1. Complete statistical analysis comparing phosphorylation of different sites of HEC1 among AURKs deficient oocytes. One-way ANOVA for each phosphorylation site.
